# Bayesian Parameter Identification for Turing Systems on Stationary and Evolving Domains

**DOI:** 10.1007/s11538-018-0518-z

**Published:** 2018-10-11

**Authors:** Eduard Campillo-Funollet, Chandrasekhar Venkataraman, Anotida Madzvamuse

**Affiliations:** School of Mathematical and Physical Sciences, University of Sussex, Brighton, UK

**Keywords:** Bayesian inverse problems, Parameter identification, Inverse problems, Markov chain Monte Carlo, Reaction–diffusion, Pattern formation, Turing instability

## Abstract

In this study, we apply the Bayesian paradigm for parameter identification to a well-studied semi-linear reaction–diffusion system with activator-depleted reaction kinetics, posed on stationary as well as evolving domains. We provide a mathematically rigorous framework to study the inverse problem of finding the parameters of a reaction–diffusion system given a final spatial pattern. On the stationary domain the parameters are finite-dimensional, but on the evolving domain we consider the problem of identifying the evolution of the domain, i.e. a time-dependent function. Whilst others have considered these inverse problems using optimisation techniques, the Bayesian approach provides a rigorous mathematical framework for incorporating the prior knowledge on uncertainty in the observation and in the parameters themselves, resulting in an approximation of the full probability distribution for the parameters, given the data. Furthermore, using previously established results, we can prove well-posedness results for the inverse problem, using the well-posedness of the forward problem. Although the numerical approximation of the full probability is computationally expensive, parallelised algorithms make the problem solvable using high-performance computing.

## Introduction

Parameter identification is the problem of extracting information about the parameters of a model from the actual result of some measurement (Tarantola 2005). In this study, our mathematical models are of the form of partial differential equations (PDEs) whose variables are elements of a Banach space. The data will be a noisy measurement of the solution of the system of PDEs, whilst the parameters will be the coefficients of the mathematical model, i.e. the coefficients of the system of PDEs. Initial and boundary conditions can also be considered as parameters as well. The data may also be a functional of the solution, such as an average. Parameter identification problems are sometimes referred to as inverse problems, thus considering as a direct problem solving the system of PDEs given the parameters. Note that parameters may be functions in general. We remark that this approach applies directly to systems of ordinary differential equations as well, making the theory of interest to a wide range of fields.

There are two main approaches to solve parameter identification problems. On the one hand, one can use techniques from optimal control to find the best possible value for the parameter (see, for instance, Aster et al. (2013), Beck et al. (1985)). Here, the best possible value is defined in terms of the distance from the solution of the model to the given data. In a Banach space setting, when the data and the solution are elements of a Banach space, this corresponds to the problem of minimising the norm of the difference between the solution and the data. In general, this problem is ill-posed. A regularisation term must be added, enforcing the norm of the parameter to be minimised too, and effectively restricting the candidate parameters. Ill-posedness may come from different sources, like non-uniqueness or non-continuous dependence of the solution with respect to the data.

A second approach to parameter identification is to use Bayesian techniques (see, for instance, Stuart 2010; Kaipio and Somersalo 2006). This approach consists of a set of techniques built around Bayes’ theorem, which, roughly speaking, allows one to compute the conditional probability of an event *A* given the probability of an event *B*, in terms of the reverse conditional probability, i.e. the probability of *B* given *A*. In the parameter identification framework, this will correspond to computing the probability of the parameters given the data, in terms of the probability of the data given the parameters. Note that the latter is given by the mathematical model: when the parameters are given, the mathematical model predicts the result of the experiment, in other words the data.

In both cases, numerical algorithms are necessary to approximate the final result. In the optimal control framework, there are a range of minimisation techniques that may be used, for instance Gauss–Newton optimisation or the Levenberg–Marquardt algorithm (see Adby 2013 for a general description of these algorithms). An optimal value can be obtained from the Bayesian framework using maximum a posteriori (MAP) methods, closely related to the maximum likelihood approach. Additional methods can be used to estimate confidence intervals for the parameters. In the Bayesian framework, the problem will be to estimate a full probability distribution. In this case, usually a Markov chain Monte Carlo algorithm is used to build a Markov chain with stationary distribution equal to the target distribution (see Norris 1998; Stuart 2010; Toni et al. 2009; Battogtokh et al. 2002; Brown and Sethna 2003). This usually requires a lot of evaluations of the observation functional—the mapping from parameters to observations, i.e. from parameters to the solution of the mathematical model—solving the system of PDEs for each evaluation (Stuart 2010). Whilst this is computationally more expensive than a minimisation algorithm, the amount of useful information obtained—the full probability distribution—is larger compared to the single value that we get from the optimal control approach. Note that in both cases a parallel algorithm can be used to speed up the computations.

From the probability distribution provided by the Bayesian framework, one can extract information about the uncertainty of the parameters, thus avoiding situations where despite obtaining a value for the parameters, a more detailed study of the uncertainty shows that they are not well determined (Gutenkunst et al. 2007). In many practical situations, the exact value of the parameters is not useful in drawing conclusions. For instance, in Ashyraliyev et al. (2008) it is shown that correlations between parameters in a model for *Drosophila Melanogaster* gap gene impede the individual identification of the parameters, but it is still possible to draw conclusions about the regulatory topology of the gene network. Furthermore, the information provided by the probability distribution can be used in model selection problems (Vyshemirsky and Girolami 2008), where several mathematical models are available and the goal is to find which one fits better to the experimental data.

There are other advantages inherent to the fact that a probability distribution for the parameters is available. For example, correlations between different parameters can be observed in the joint probability distribution. These correlations can be invisible when only a value for the parameters is computed, leading to an overestimation of the uncertainty (Sutton et al. 2016). Similarly, the correlations between parameters can suggest a non-dimensionalised form of the equations to eliminate non-relevant parameters from the problem.

A drawback of the Bayesian approach is its computational cost. Many problems were not suitable for this approach a few years ago due to the lack of computational power (SIAM Working Group on CSE 2001). Recent advances in HPC scientific computing now offer new avenues to perform highly efficient, robust and fast computations within the timescales necessitated by experimental manipulations.

Parameter identification problems arise often in many areas of research such as biology, biomedicine, chemistry, material sciences, mathematics, statistics. Typically, a mathematical model needs to be fitted to experimental data. Only in a few cases it is possible to perform an analytical parameter identification, see, for instance, Friedman and Reitich (1992). Note also that parameters are not always directly measurable from experiments, or have a defined physical meaning. Hence, parameter identification through the proposed approach might help guide experimentalists in identifying regions of interest to which parameters belong as well as helping them sharpen their experimental hypotheses.

The optimal control approach is applied in Ashyraliyev et al. (2009) to systems of ordinary differential equations modelling enzyme kinetics. In this case, confidence regions may be computed using the second derivatives of the cost functional that is being minimised. Since this only provides local information around the optimal point, the confidence regions computed with this method tend to be rather large. The optimal control approach has also been applied to geometric evolution problems for cell motility, and to reaction–diffusion–transport systems (Garvie et al. 2010; Croft et al. 2015; Blazakis et al. 2015; Portet et al. 2015; Yang et al. 2015; Stoll et al. 2016).

Bayesian techniques are common for parameter fitting in statistics (see, for example, Ma and Leijon 2011; Fan et al. 2012; Ma et al. 2015). The Bayesian approach is also used in many fields for forward models governed by PDEs (see Stuart (2010) and the references therein), for example inverse problems in subsurface flow (Iglesias et al. 2014) and 3D modelling of the cardiovascular system (Perdikaris and Karniadakis 2016). This approach is based on using numerical methods to solve the PDE, although in some cases it is possible to represent the solution operator via basis function expansion (Xun et al. 2013).

Turing systems are a family of reaction–diffusion systems introduced by Turing (1952). Originally intended to model morphogenesis, they have since found many applications in mathematical biology and other fields (Lacitignola et al. 2017; Das 2017; Wang et al. 2016; Hu et al. 2015; Guiu-Souto and Muñuzuri 2015). The main feature of these models is that small perturbations of the homogeneous steady states may evolve to solutions with non-trivial patterns (Murray 2011, 2013). An example of such a model is the Schnakenberg system, also known as the activator-depleted substrate model (Gierer and Meinhardt 1972; Prigogine and Lefever 1968; Schnakenberg 1979). This model has been widely studied, both analytically and numerically (Garvie et al. 2010; Venkataraman et al. 2012; Madzvamuse et al. 2005). Note that for some reaction–diffusion systems with classical reaction kinetics the Turing space can be obtained analytically, thereby offering us a bench-marking example for our theoretical and computational framework (see, for instance, Murray 2013).

The parameter identification for Turing systems, using Turing patterns as data, was studied in Garvie et al. (2010), both with Schnakenberg kinetics and with Gierer–Meinhardt kinetics, but using an optimal control approach. Garvie and Trenchea (2014) also used an optimal control approach to identify space–time distributed parameters in a system with Gierer–Meinhardt kinetics. In contrast, here we use a Bayesian framework that, in particular, allows the consistent computation of credible regions for the estimated parameters. To the best of our knowledge, this is the first study to address the inverse problem for Turing patterns in the Bayesian framework, and in particular, in growing domains. Uzunca et al. (2017) studies a convective FitzHugh–Nagumo system which is similar to the system arising in growing domains and identifies finite-dimensional parameters using optimisation techniques.

Our first goal is to identify a time-dependent growth function for a reaction–diffusion system posed on a one-dimensional growing domain. For this case, the rest of the model parameters are considered known and fixed. This example illustrates an application of our approach to infinite-dimensional parameter identification. Our second example is that of parameter identification in a finite-dimensional framework where we consider the reaction–diffusion system posed on a stationary two-dimensional domain. For both examples, we use synthetic patterns—computer generated—based on previous works using a fixed set of parameters (Garvie et al. 2010; Madzvamuse et al. 2010).

## Parameter Identification

The main ingredients of a parameter identification problem are the data (the measurements from the experiment), the parameters and the mathematical model. In accordance with the functional analytic framework for the treatment of PDE models, the data and the parameters are modelled as elements of suitable Banach spaces. To fix the notation, let *U* and *Y* be Banach spaces, let $y\in Y$ be the data, and let $p \in U$ be the parameter. Note that in general *p* is a vector of parameters. When one fixes a value for the parameter *p*, one can solve the equations of the model to find the solution associated with this parameter. This process is known as the solution of the forward problem; in contrast, the problem of parameter identification is an inverse problem. The solution of the forward problem is formalised with a mapping $G:U\rightarrow Y$ such that a parameter $p\in U$ is mapped to the solution of the model with parameter *p*. Since *G* maps parameters to the measurement space, *G* is called the observation operator. *G* is well defined as long as for each possible parameter, there exists a unique solution to the mathematical model; otherwise, *G* might not be defined in *U* or might be multivalued.

Noise that is present in the data is naturally modelled by means of a probability distribution. Let ${\mathbb {Q}}_0$ be a probability distribution. Let $\eta$ be a realisation of ${\mathbb {Q}}_0$. With this notation at hand, our measurement can be expressed as

1 $$\begin{aligned} y = G(p) + \eta . \end{aligned}$$

The Bayesian approach to parameter identification assumes implicitly that a probability distribution models the state of knowledge about a quantity (Tarantola 2005). If the quantity is known with infinite precision then the probability distribution will be concentrated at one point—a Dirac delta distribution. The less we know, the more spread the distribution will be.

Equation (1) expresses the relation between the probability distribution of the data *y*, the parameters *p* and the noise $\eta$. In other words, (1) connects our knowledge about the different ingredients of the parameter identification problem. Note that the mathematical model, encoded in *G*, is assumed to be known; our interest is to find information about the parameters of a given model, not to find the model itself, although dummy parameters could be used for model selection.

From the modelling point of view, Eq. (1) is well defined as long as the data *y* lie in a Banach space. Some systems may require a more general framework, for instance if the data lie in a manifold or in a metric space, where the operation “$+$” may not be defined. The Banach space setting allows us to prove rigorous results on the parameter identification problem, and it is suitable for the numerical approximation of the solution (Stuart 2010; Dashti and Stuart 2013; Cotter et al. 2013). In terms of applicability, in some situations the task of finding the noise distribution is not trivial (Kaipio and Somersalo 2007; Huttunen and Kaipio 2007).

The parameter identification problem can be now stated as follows: find the probability distribution of the parameter *p* given the data *y*. This probability distribution is called the posterior and is denoted by ${\mathbb {P}}(p|y)$, or ${\mathbb {P}}^y(p)$. All the information available about the parameter *p* is encoded in the posterior probability distribution.

We can now apply Bayes’ theorem to (1). Let $\mu ^y$ be the probability density function of ${\mathbb {P}}(p|y)$. The PDF of ${\mathbb {P}}(y|p)$ is given by $\rho (y-G(p))$, where $\rho$ is the PDF of the noise distribution ${\mathbb {Q}}_0$. Denote by $\mu _0$ the PDF of the prior distribution. From Bayes’ theorem we obtain (Stuart 2010)

$$\begin{aligned} \mu ^y(p) \propto \rho (y-G(p))\mu _0(p). \end{aligned}$$

Bayes’ theorem can be generalised to an infinite-dimensional setting. The formulation is more technical but analogous to the finite-dimensional case described above. With the general version of the theorem, the Bayesian approach to parameter identification can be applied to infinite-dimensional problems. Let ${\mathbb {P}}_0$ be the prior distribution. The following result generalises the Bayes’ theorem to infinite-dimensional settings (Stuart 2010; Dashti and Stuart 2013).

### Theorem 1

(Bayes’ theorem) Assume that the potential $\varPhi : U \times Y \longrightarrow {\mathbb {R}}$ is measurable with respect to the product measure ${\mathbb {P}}_0 \times {\mathbb {Q}}_0$, and assume that

$$\begin{aligned} Z := \int _U \exp \left( -\varPhi (p;y) \right) {\mathbb {P}}_0(\mathrm {d}p) > 0. \end{aligned}$$

Then, the conditional distribution ${\mathbb {P}}(p|y)$, denoted by ${\mathbb {P}}^y(p)$, exists, and it is absolutely continuous with respect to the prior distribution ${\mathbb {P}}_0$. Furthermore,

$$\begin{aligned} \frac{\mathrm {d}{\mathbb {P}}^y}{\mathrm {d}{\mathbb {P}}_0} = \frac{1}{Z} \exp \left( - \varPhi (p;y) \right) . \end{aligned}$$

### Proof

See (Dashti and Stuart 2013, theorem 3.4). $\square$

Note that the potential $\varPhi$ typically corresponds to the negative log-likelihood. Furthermore, since the data *y* are fixed in the applications, we drop the dependency on *y*; that is, we write $\varPhi (p)$ instead of $\varPhi (p;y)$.

Using only properties of the forward problem, the following proposition (see Stuart 2010) guarantees that the parameter identification problem is well-posed: the posterior distribution exists, and it depends continuously on the data *y*. Denote by $B(0,r)\subset U$ the ball with centre at the origin and radius *r*, and by $\Vert \cdot \Vert _\varSigma$ the Euclidean norm with weight $\varSigma$.

### Proposition 1

(Well-posedness of the Bayesian inverse problem) Assume that ${\mathbb {Q}}_0$ is a Gaussian distribution with covariance $\varSigma$ and that the potential is $\varPhi (p;y) = \Vert y - G(p)\Vert _\varSigma ^2$. Assume that the observation operator *G* satisfies the following conditions.

(i) For all $\varepsilon$, there exists $M\in {\mathbb {R}}$ such that for all $p\in U$,
  $$\begin{aligned} \Vert G(p)\Vert _\varSigma \le \exp \left( \varepsilon \Vert p\Vert _U^2 + M \right) . \end{aligned}$$
(ii) For all $r>0$, there exists a *K* such that for all $p_1,p_2\in B(0,r)$,
  $$\begin{aligned} \Vert G(p_1) - G(p_2)\Vert _\varSigma \le K\Vert p_1 - p_2\Vert _U. \end{aligned}$$

Then, the distribution ${\mathbb {P}}^y(p)$ defined in Theorem 1 exists and depends continuously on the data *y*.

### Proof

See (Stuart 2010, theorem 4.2). $\square$

Note that the first condition is a bound on the growth of the solution with respect to the parameter *p*, whilst the second condition is the Lipschitz continuity of the observation operator. The first condition is not necessary to obtain well-posedness if the parameter space *U* is finite-dimensional.

We remark that in order to solve a parameter identification problem with infinite-dimensional parameters, we require that the problem is well-posed in the infinite-dimensional setting, according to Theorem 1 and Proposition 1. Later, for the numerical approximation of the problem, we will discretise the parameter space, and the problem will become finite-dimensional. However, it is not enough to show that the problem is well-posed in the finite-dimensional setting. The discrete solution approximates the infinite-dimensional solution, and therefore, we need to know that we are approximating a meaningful object in the infinite-dimensional setting. See Dashti and Stuart (2013) for some results on the convergence with respect to the discretisation.

Bayes’ theorem characterises the posterior probability distribution, but involves the normalisation constant *Z* (see Proposition 1). *Z* is defined as an integral over the entire parameter space, with respect to the prior measure, and it is computationally expensive to evaluate even for finite-dimensional problems. An alternative is to not compute the distribution, but to produce samples from it.

Markov chain Monte Carlo (MCMC) methods are a family of methods that produce a Markov chain with a given distribution (Norris 1998). For the problem at hand, the target distribution is the posterior. Therefore, for each new state of the Markov chain, we obtain a sample of the posterior distribution.

MCMC methods are robust but slow. The distribution of the Markov chain converges, under general conditions, to the target distribution, but long chains are necessary to obtain good approximations (Norris 1998). Also, the methods are inherently sequential. Furthermore, in the parameter identification framework, evaluation of the acceptance probability typically involves, at least, one evaluation of the potential $\varPhi$, and in consequence, one evaluation of the observation operator *G*. This is an expensive operation in terms of computing time in cases where the model is a system of PDEs because it entails solving the system.

To overcome these difficulties we shall use a parallel Metropolis–Hastings algorithm (Calderhead 2014; Tjelmeland 2004). The key idea is to generate an *N-dimensional* Markov chain such that its distribution is *N* copies of the target distribution. This can be done in a way that allows parallel evaluations of the potential $\varPhi$.

Let $x_k$ be the current state of the Markov chain, and let $k(x_k,x)$ be a proposal kernel—the probability to propose a state *x* if the present state is $x_k$. For ease of presentation assume that the proposal kernel is symmetric, i.e. $k(x_k,x)=k(x,x_k)$. Let $a(x_k,x)$ be the probability of accepting a new state *x* if the present state is $x_k$.

The choice of the proposal kernel *k* is critical to the performance of the algorithm. Furthermore, the choice of the acceptance probability *a* depends on the proposal kernel in order to ensure that the Markov chain is reversible with respect to the target probability. Reversibility implies, in turn, that the Markov chain preserves the target probability. We refer to Tierney (1998) for details on the choice of *a*, in general, and to Cotter et al. (2013) for the case of infinite-dimensional states.

In the present work, we restrict ourselves to a simple proposal kernel. Given the prior distribution ${\mathbb {P}}_0$, take the proposal kernel *k* satisfying

$$\begin{aligned} k(x_k,\cdot ) \sim {\mathbb {P}}_0(\cdot ). \end{aligned}$$

Together with the acceptance probability given by

2 $$\begin{aligned} a(x_k,x) = \min \left\{ 1,\exp \left( \varPhi (x_k) - \varPhi (x) \right) \right\} , \end{aligned}$$

this choice corresponds to the standard Metropolis–Hastings method known as independence sampler. We refer to Cotter et al. (2013) for other choices suitable for infinite-dimensional problems. Note that the acceptance probability in this case, as well as for many other popular choices, depends only on the potential $\varPhi$.

Although the independence sampler is useful for a general exploration of the parameter space, it does not offer any tunable parameters, and therefore, it may work poorly when the values of the potential $\varPhi$ have significant differences depending on where it is evaluated.

Another popular sampler is the preconditioned Crank–Nicolson (pCN). For a Gaussian prior with mean 0 and covariance $\varGamma$, the proposal kernel for the pCN is given by

$$\begin{aligned} k(x_k,x) \sim {\mathcal {N}}( ( 1 - \beta )^{\frac{1}{2}} x_k, \beta ^2 \varGamma ), \end{aligned}$$

where ${\mathcal {N}}(m,C)$ denotes a Gaussian distribution with mean *m* and covariance *C*, and $\beta \in [0,1]$. The acceptance probability is the same as in the independence sampler, (2). With the pCN, we can adjust the variance of the proposals by tuning the parameter $\beta$. The proposals account for the local information provided by the current state $x_k$. For $\beta =1$, we recover the independence sampler. Taking smaller values of $\beta$ entails that the samples are closer to the current state and the acceptance probability increases.

Both independence sampler and pCN are suitable for infinite-dimensional parameters. Cotter et al. (2013) consistently derive these and other proposal kernels by discretising a Langevin-type stochastic differential equation.

We note that for some problems of interest, the evaluation of the potential $\varPhi$ is computationally intensive, and therefore, proposal kernels that require evaluations of quantities like $\nabla \varPhi$ may not be feasible.

In the standard Metropolis–Hastings method, given a current state $x_k$, we generate a proposal *x* from the proposal kernel. We accept the new proposal with probability $a(x_k,x)$, i.e. $x_{k+1} = x$ with probability $a(x_k,x)$, and $x_{k+1} = x_k$ otherwise. Note that the evaluation of $a(x_k,x)$ involves the evaluation of $\varPhi (x)$, which in turn involves the evaluation of the observation operator *G*. Since this evaluation of *G* is an expensive operation, the aim of the parallel Metropolis–Hastings method is to evaluate $\varPhi$ in parallel for many proposals.

The parallel Metropolis–Hastings algorithm goes as follows. We generate *N* new proposals $\{x^j\}_{j=1}^N$ from the proposal kernel $k(x_k,\cdot )$. Take $x^0 = x_k$. Then, in parallel, we evaluate the potentials $\varPhi (x^j)$, $j=1\ldots N$. Note that this will be done by *N* instances of the PDE solver running in parallel, but the solver itself need not be parallel. Parallelising the solver for the model will reduce the time spent solving the model system and therefore is, in some cases, worth implementing. With the values of $\varPhi (x^j)$ at hand, the acceptance probability of each proposal is computed by finding the stationary distribution of a Markov chain with $N+1$ states, given by the transition matrix

$$\begin{aligned} A(i,j) = \left\{ \begin{aligned} \frac{1}{N}a(x^i,x^j)&\quad \text { if } j\ne i, \\ 1 - \sum _{j\ne i} A(i,j)&\quad \text { if } j=i. \end{aligned} \right. \end{aligned}$$

Finally, we sample *N* times from the stationary distribution to produce *N* new states (see Algorithm 1). Note that this approach is non-intrusive, in the sense that it does not require modifications of the solver of the PDEs.
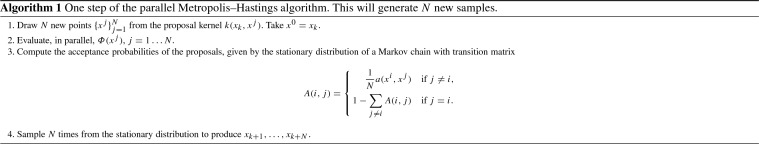


There are many other sampling methods to approximate a probability distribution characterised by the Bayes’ theorem. The parallelised Metropolis–Hastings method presented above is suitable when a PDE solver for the mathematical model is already available, and the only interest is in speeding up the computations using a parallel HPC environment, without doing any changes to the solver. In other words, it is a parallel sampling algorithm that works unintrusively with a given PDE solver to evaluate the potential $\varPhi$. Furthermore, the Metropolis–Hastings methods work well for problems with infinite-dimensional parameters (Stuart 2010; Dashti and Stuart 2013).

We remark that in this context, we assume that we can explicitly compute the potential $\varPhi$; that is, we can compute the likelihood. This is a consequence of (1) and allows us to incorporate in the modelling the information available on the experimental noise. In other problems, specially when the noise distribution is completely unknown, or when the likelihood cannot be computed in general, approximate Bayesian computation can be more suitable. See Ross et al. (2017) for an example of application of approximate Bayesian computation in mathematical biology.

The method described in this study has been implemented in Python, using the module Scipy (Jones et al. 2001) and the module *multiprocessing* from the Python Standard Library. The evaluation of the potential $\varPhi$ can be implemented using a different language and then invoked from the Python code. In this paper, we use Python also for the evaluation of $\varPhi$; see Sect. 3 for details.

### HPC Computations

For each of the examples shown in Sect. 3, we generate 10 Markov chains for a total of approximately $10^6$ samples. The mean and the correlation of each chain are examined and are used to decide the burn-in—the fraction of the chain discarded due to the influence of the initial value. The burn-in fraction is determined by checking the convergence of the mean for each chain. Finally, the chains are combined in a big set that we use to generate the plots.

All the results shown are generated using the local HPC cluster provided and managed by the University of Sussex. This HPC cluster consists of 3000 computational units. The specifications of the computational units are AMD64, x86_64 or 64 bit architecture, made up of a mixture of Intel and AMD nodes varying from 8 cores up to 64 cores per node. Each unit is associated with 2 GB memory space. Most of the simulations in this paper are executed using 8–48 units. The wall-clock computation time for one chain ranged from 1 to 4 days, and the CPU time ranged from 10 to 35 days for one chain. It must be noted that there are two levels of parallelisation: first the algorithm is parallelised and uses 8 of the available cores, and then many instances of the algorithm run at the same time to produce independent chains. We remark that we produce independent chains in order to test the convergence of the algorithm.

## Turing Systems

We consider infinite-dimensional (as well as finite-dimensional) parameter identification for a well-known mathematical model, a reaction–diffusion system on evolving and stationary domains. As mentioned earlier, we take the Schnakenberg kinetics (Gierer and Meinhardt 1972; Prigogine and Lefever 1968; Schnakenberg 1979) for illustrative purposes.

### Reaction–Diffusion System Posed on Uniform Isotropic Evolving Domains

Let $$\varOmega _t \subset {{\mathbb {R}}}^m$$$$(m=1,2)$$ be a simply connected bounded evolving domain for all time $$t \in I=[0,t_F]$$, $$t_F > 0$$ and $${\partial \varOmega _t}$$ be the evolving boundary enclosing $$\varOmega _t$$. Also let $$ {{\varvec{u}}} = \left( u \left( {{\varvec{x}}} (t),t \right) , v \left( {{\varvec{x}}} (t),t \right) \right) ^T$$ be a vector of two chemical concentrations at position $${{\varvec{x}}} (t) \in \varOmega _t \subset {{\mathbb {R}}}^m$$. The growth of the domain $$\varOmega _t$$ generates a flow of velocity $${{\varvec{v}}}$$. For simplicity, let us assume a uniform isotropic growth of the domain defined by $$ {{\varvec{x}}} (t) = \rho (t) {{\varvec{x}}} (0)$$, where $${{\varvec{x}}}(0) \in \varOmega _0$$ is the initial domain and $$\rho (t)\in C^1(0,1)$$ is the growth function (typically exponential, linear or logistic). We further assume that the flow velocity is identical to the domain velocity $$ {{\varvec{v}}} : = \frac{d {{\varvec{x}}}}{dt}.$$ The evolution equations for reaction–diffusion systems can be obtained from the application of the law of mass conservation in an elemental volume using Reynolds transport theorem. Since the domain evolution is known explicitly, a Lagrangian mapping from an evolving to a stationary reference initial domain yields the following non-dimensional reaction–diffusion system with time-dependent coefficients (Crampin et al. 2002; Mackenzie and Madzvamuse 2011; Madzvamuse and Maini 2007; Madzvamuse et al. 2010, 2016)

3 $$\begin{aligned} {\left\{ \begin{array}{ll} {\left\{ \begin{array}{ll} u_t +\frac{m {\dot{\rho }} (t)}{\rho (t)} u= \frac{1}{\rho ^2 (t)} \Delta u + \gamma f(u,v), \\ v_t +\frac{m {\dot{\rho }} (t)}{\rho (t)} v= \frac{d}{\rho ^2 (t)} \Delta v + \gamma g(u,v), \end{array}\right. } \quad {{\varvec{x}}} \in \varOmega _0, \\ \\ {{\varvec{n}}} \cdot \nabla u= {{\varvec{n}}} \cdot \nabla v =0,\; {{\varvec{x}}}\,\text {on}\,\partial \varOmega _0, \\ \\ u({{\varvec{x}}},0)= u_0({{\varvec{x}}}),\; \text {and} \; v({{\varvec{x}}},0)=v_0({{\varvec{x}}}),\; {{\varvec{x}}}\,\text {on}\;\varOmega _0, \end{array}\right. } \end{aligned}$$

where $\Delta$ is the Laplace operator on domains and volumes, *d* is the ratio of the diffusion coefficients, and ${\dot{\rho }}:=\frac{d \rho }{dt}$. Here, ${{\varvec{n}}}$ is the unit outward normal to $\varOmega _t$. Initial conditions are prescribed through non-negative bounded functions $u_0 ({{\varvec{x}}})$ and $v_0 ({{\varvec{x}}})$. In the above, *f*(*u*, *v*) and *g*(*u*, *v*) represent nonlinear reactions and these are given by the activator-depleted kinetics (Crampin et al. 1999; Gierer and Meinhardt 1972; Prigogine and Lefever 1968; Schnakenberg 1979)

4 $$\begin{aligned} f(u,v) = a - u + u^2 v, \quad \text {and} \quad g(u,v) = b - u^2 v. \end{aligned}$$

To proceed, let us fix the parameters $a, b, d, \gamma \ge 0$, and use the Bayesian approach to identify the domain growth rate function $\rho (t)$, assuming $\rho (0) = 1$, $\rho (t)> 0$ for $t>0$ and that the domain size at the final time, $\rho (T)$, is known.

Well-posedness results for the system of Eq.  (3), as well as stability results, can be found in Venkataraman et al. (2012). Furthermore, the positivity of solutions is established. These results ensure that the conditions in Proposition 1 are satisfied, and thus, we can conclude the well-posedness of the parameter identification problem.

#### Proposition 2

(Well-posedness of the parameter identification problem) Let *G* be the observation operator associated with the reaction–diffusion system (3) with reaction kinetics (4). Assume that *a*, *b*, *d*, $\gamma > 0$, and $\rho \in C^1(0,T)$, $\rho >0$. Then, the conditions in Proposition 1 are satisfied, and thus the parameter identification problem is well-posed.

#### Proof

The result follows from the analysis in Venkataraman et al. (2012), where well-posedness for problem (3) is shown. In particular, the first condition in Proposition 1 is a consequence of the a priori estimates for the solution. More precisely, the estimates in Venkataraman et al. (2012) show in particular that the $L^2$-norm of the solution to (3) is controlled by the square of the $L^2$-norm of the domain growth, a bound tighter than the one required in Proposition 1. As noted, this bound is only required for parameters in an infinite-dimensional space. $\square$

Although this example is synthetic, it is biologically plausible. First, we generate the data by adding noise to a numerical solution of the system, but the parameter identification problem would be analogous in the case of experimental data (see, for instance, Vigil et al. (1992) for an experiment that produces Turing patterns similar to the patterns that we use as data in Example 2).

Since the noise follows a Gaussian distribution, we evaluate the potential $\varPhi$ according to Proposition 1. Let $\tilde{G}$ be the observation operator associated with the numerical solver that maps a parameter *p* to the numerical approximation of the solution of (3). Let *y* be the data, and let $\varSigma$ be the covariance of the noise. Then,

$$\begin{aligned} \varPhi (p;y) = \Vert \tilde{G}(p) - y\Vert ^2_\varSigma . \end{aligned}$$

#### Example 1: Infinite-Dimensional Parameter Identification

Without any loss of generality, we restrict our first example to the one-dimensional case ($m=1$) where we fix model parameters with standard values in the literature as (Murray 2013)

$$\begin{aligned} a =0.1, \quad b= 0.9, \quad d= 10, \quad \gamma = 1000. \end{aligned}$$

We want to identify the growth function $\rho (t)$ given synthetic data generated from two different growth profiles defined by the exponential and logistic functions

5 $$\begin{aligned} \rho _{\exp } (t) = \exp (0.001t),\quad \text {and} \quad \rho _{\log } (t) = \frac{\exp (0.01t)}{1+\frac{\exp (0.01t)-1}{\exp (0.006)}}. \end{aligned}$$

We fix the final time $T=600$. Note that the size of the domain at time *T* is the same in both cases.

Initial conditions are taken as small fixed perturbations around the homogeneous steady state, $(u_*,v_*)=\left( a+b, \frac{b}{(a+b)^2} \right)$, and these are given by

6 $$\begin{aligned} u_0(x) = 1 + 0.005\sum _{k=1}^9 \sin (k\pi x),\quad \text {and} \quad v_0(x) = 0.9 + 0.005\sum _{k=1}^9 \sin (k\pi x). \end{aligned}$$

We solve the system using the finite difference method both in space and in time. The system of partial differential equations is solved on the mapped initial unit square domain using a finite difference scheme. Similar solutions can be obtained by employing finite element methods for example or any other appropriate numerical method. The algebraic linear systems are solved using a conjugate gradient method from the module *SciPy* (Jones et al. 2001).

The time-stepping scheme is based on a modified implicit–explicit (IMEX) time-stepping scheme where we treat the diffusion part and any linear terms implicitly and use a single Picard iterate to linearise nonlinear terms (Ruuth 1995; Madzvamuse 2006; Venkataraman et al. 2012). This method was analysed in Lakkis et al. (2013) for finite element discretisation. It is a first-order, semi-implicit backward Euler scheme, given by

7 $$\begin{aligned} \begin{aligned} \frac{u^{n+1} - u^n}{\tau } + \frac{{\dot{\rho }}^n}{\rho ^n} u^{n+1}&= \frac{1}{\rho ^{2n}} \Delta _h u^{n+1} + \gamma (a - u^{n+1} + u^n u^{n+1} v^n), \\ \frac{v^{n+1} - v^n}{\tau } + \frac{{\dot{\rho }}^n}{\rho ^n} v^{n+1}&= \frac{d}{\rho ^{2n}} \Delta _h u^{n+1} + \gamma (b - (u^n)^2 v^{n+1}), \end{aligned} \end{aligned}$$

where $\Delta _h$ is the standard 3-point (or 5-point) stencil finite difference approximation of the Laplacian operator in 1-D (or 2-D), respectively, with Neumann boundary conditions. The parameters of the solver are $h=10^{-2}$ and $\tau =10^{-4}$ for all the computations.

**Fig. 1:**
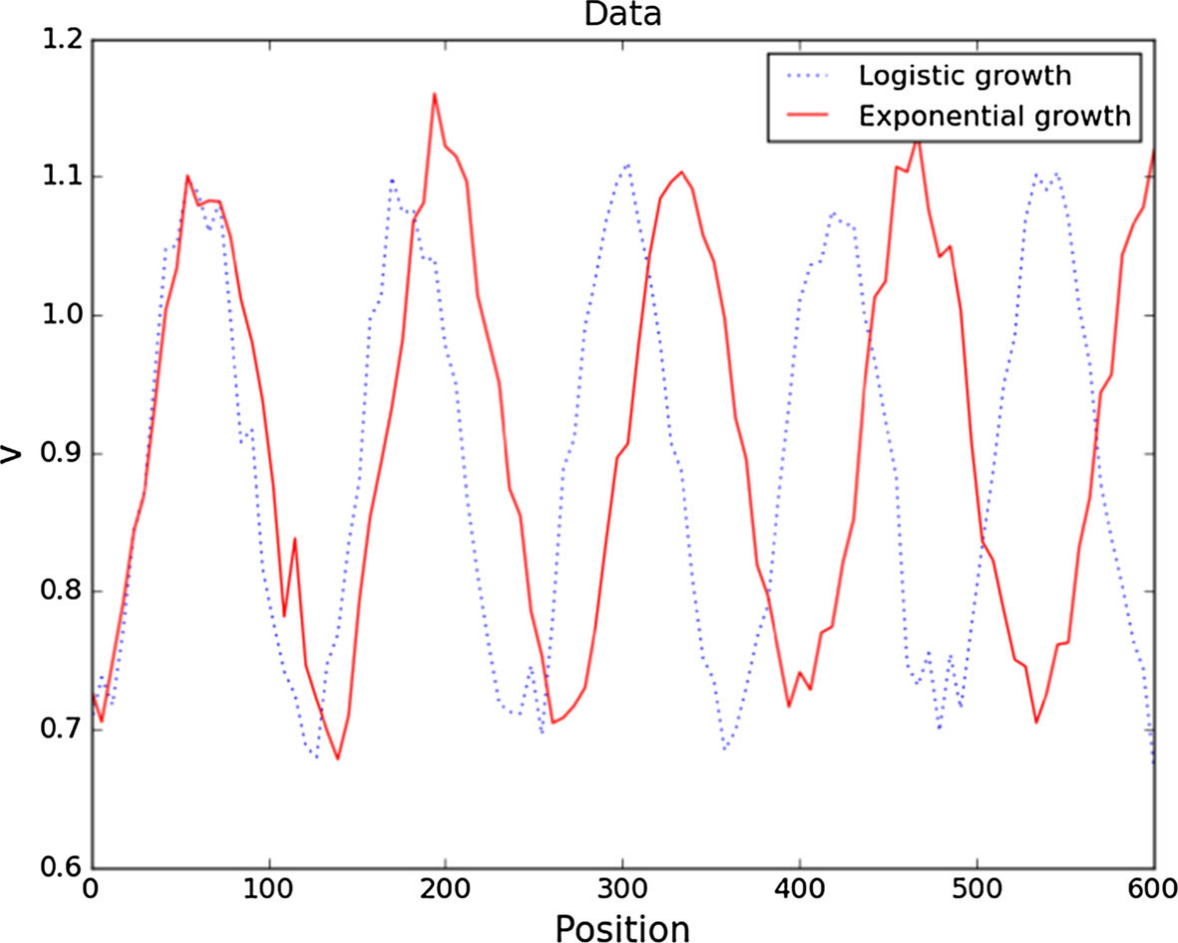
Synthetic data for the reaction–diffusion system (3) with reaction kinetics (4) on a one-dimensional growing domain with exponential and logistic growth functions. The figure depicts only the *v*-component of the solution; the *u*-component is 180 degrees out of phase (Color figure online)

We remark that our aim here is to illustrate the applicability of the Bayesian approach and the Monte Carlo methods for parameter identification to problems emanating from mathematical biology. More sophisticated solvers can be used and will improve the computational time (Venkataraman et al. 2013). The synthetic data are generated by solving the reaction–diffusion system (3) with reaction kinetics (4) up to the final time $T=600$, and then perturbing the solution with Gaussian noise with mean zero and standard deviation equal to 5% of the range of the solution. The data are illustrated in Fig. 1.

**Fig. 2:**
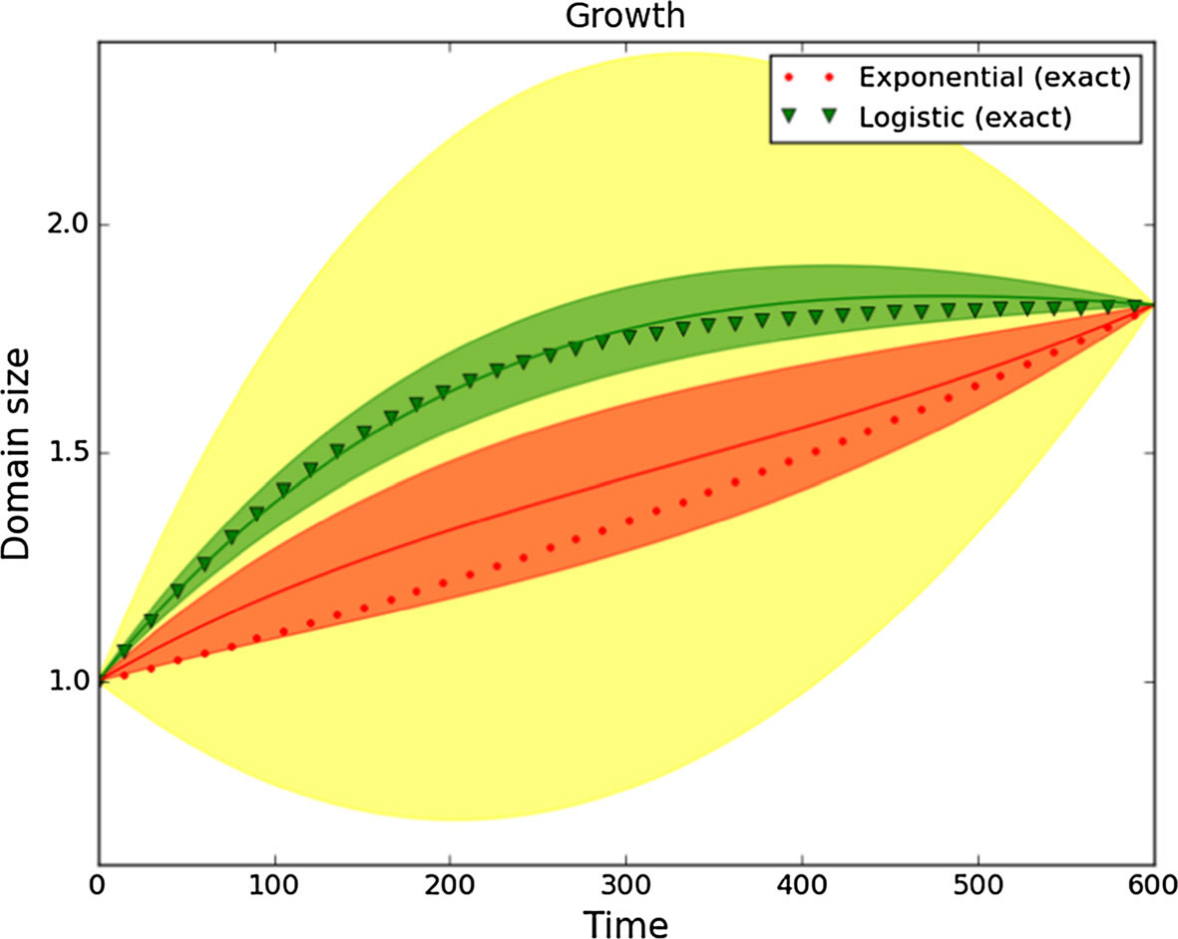
Regions that contain $95\%$ of the samples of the prior (light colour, larger region) and the posteriors for exponential growth (darker colour, bottom region) and logistic growth (darker colour, upper region). The exact growth used to generate the data is traced with triangles (logistic) and circles (exponential). The solid lines are the growth rates computed using the mean of each posterior distribution (Color figure online)

In order to identify a time-dependent parameter $\rho (t)$, we approximate it on a finite-dimensional space by using a polynomial of degree four, with only three degrees of freedom. The coefficients of order zero and four are fixed in order to satisfy the conditions for $\rho (t)$ at times $t=0$ and $t=T$, respectively. The priors for the coefficients of the polynomial approximation of $\rho (t)$ are Gaussian distributions, adjusted to ensure that $95\%$ of the samples lie in the large shaded region in Fig. 2 (yellow in colour version).

The same prior is used for both the logistic and the exponential growth data sets. In Fig. 2 we depict the regions where 95% of the samples from the posteriors lie, and also the region where 95% of the samples of the prior lie. Observe that we could also plot the credible regions for the coefficients of the finite-dimensional approximation of $\rho$, but it is more difficult to visualise the result from it. We compute these credible regions as highest posterior density regions; that is, the smallest region of the parameter space has probability 0.95 with respect to the posterior distribution.

For the final time $T=600$, we find that the region corresponding to the parameters identified from the exponential growth rate and the region corresponding to the logistic growth rate are completely separated: we can distinguish the type of growth from the solutions at the final time *T*.

#### Example 2: Finite-Dimensional Parameter Identification

Next, we demonstrate the applicability of our approach to identifying credible regions for parameters which are constant and not space nor time dependent. This is an often encountered problem in parameter identification. We will again use the reaction–diffusion system (3) with reaction kinetics (4) in the absence of domain growth, i.e. $\rho (t)=1$ for all time. Our model system is therefore posed on a stationary domain, for the purpose of demonstration, and we assume a unit square domain. We seek to identify *a*, *b*, *d* and $\gamma$. For ease of exposition, we will seek parameters in a pair-wise fashion.

**Table 1:** Exact values of the parameters used to generate the noisy data shown in Fig. 3

Parameter	Value
*a*	0.126779
*b*	0.792366
*d*	10
$\gamma$	1000

Our “experimental data” are measurements of the steady state of the system. We fix the initial conditions as a given perturbation of the spatially homogeneous steady state given by $(a+b,\frac{b}{(a+b)^2})$. For the values of the parameters given in Table 1, the initial conditions are given by

8 $$\begin{aligned} \begin{aligned} u_0(x,y)&= 0.919145 + 0.0016 \cos (2\pi (x+y)) + 0.01\sum _{j=1}^8 \cos (2\pi j x), \\ v_0(x,y)&= 0.937903 + 0.0016 \cos (2\pi (x+y)) + 0.01\sum _{j=1}^8 \cos (2\pi j x). \end{aligned} \end{aligned}$$

**Fig. 3:**
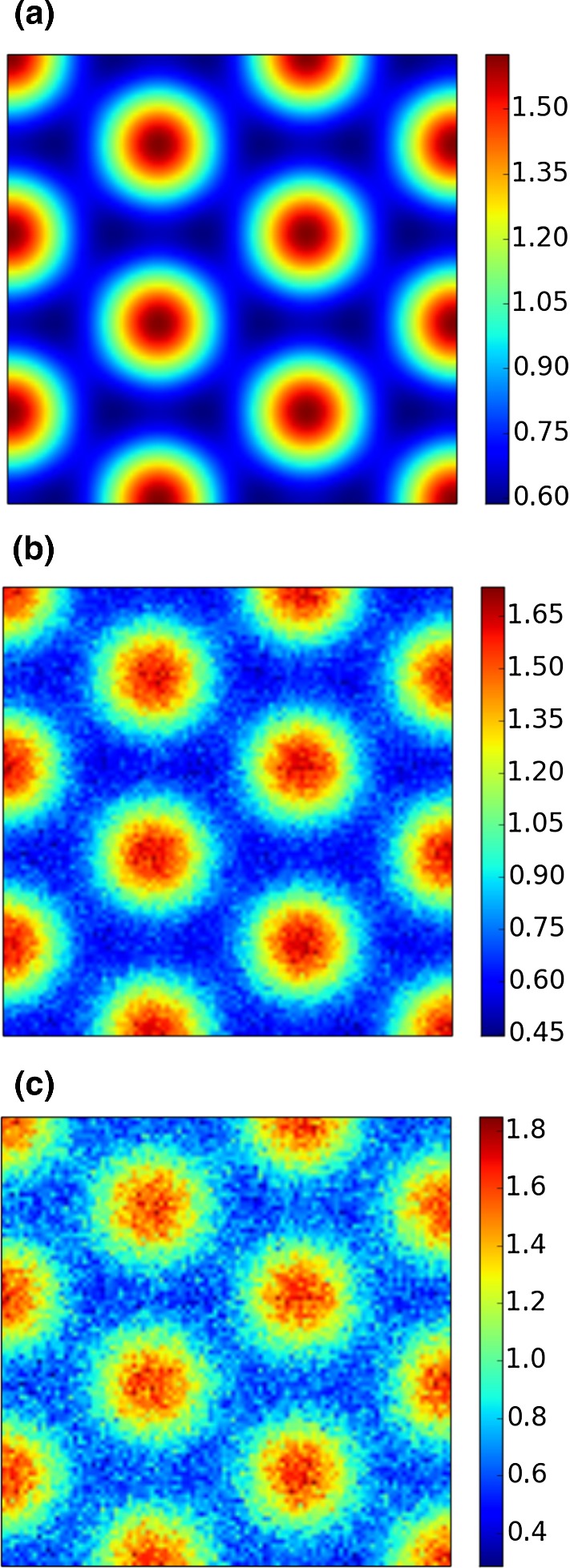
The *u*-component of the solution to the Schnakenberg system (Gierer and Meinhardt 1972; Prigogine and Lefever 1968; Schnakenberg 1979), without noise (**a**), and with added Gaussian noise with mean zero and standard deviation 5% (**b**) and 10% (**c**) of the range of the solution. Solutions of the *v*-component are 180 out of phase with those of *u*, and as such, their plots are omitted (Color figure online)

We let the system evolve until the $L_2$-norm of the discrete time derivative is smaller than a certain threshold. For the numerical experiments presented here, the threshold is $10^{-5}$. At that point, we assume that a spatially inhomogeneous steady state has been reached. We record the final time *T* and save the solution. To confirm that the solution is indeed stationary, we keep solving the system until time $t=2T$ and check that the difference between the solutions at time *T* and 2*T* is below the threshold. To generate our synthetic measurement, we add Gaussian noise to the solution, as illustrated in Fig. 3.

This synthetic experiment is a situation similar to what one will face in an actual experiment, although some assumptions, in particular the fixed known initial conditions, are not realistic. A detailed study of the dependence of the solution with respect to initial conditions will be necessary to drop this assumption. Alternatively, the initial conditions could be included as a parameter to identify.

#### Case 1: Credible Regions for *a* and *b* with Little Knowledge

**Fig. 4:**
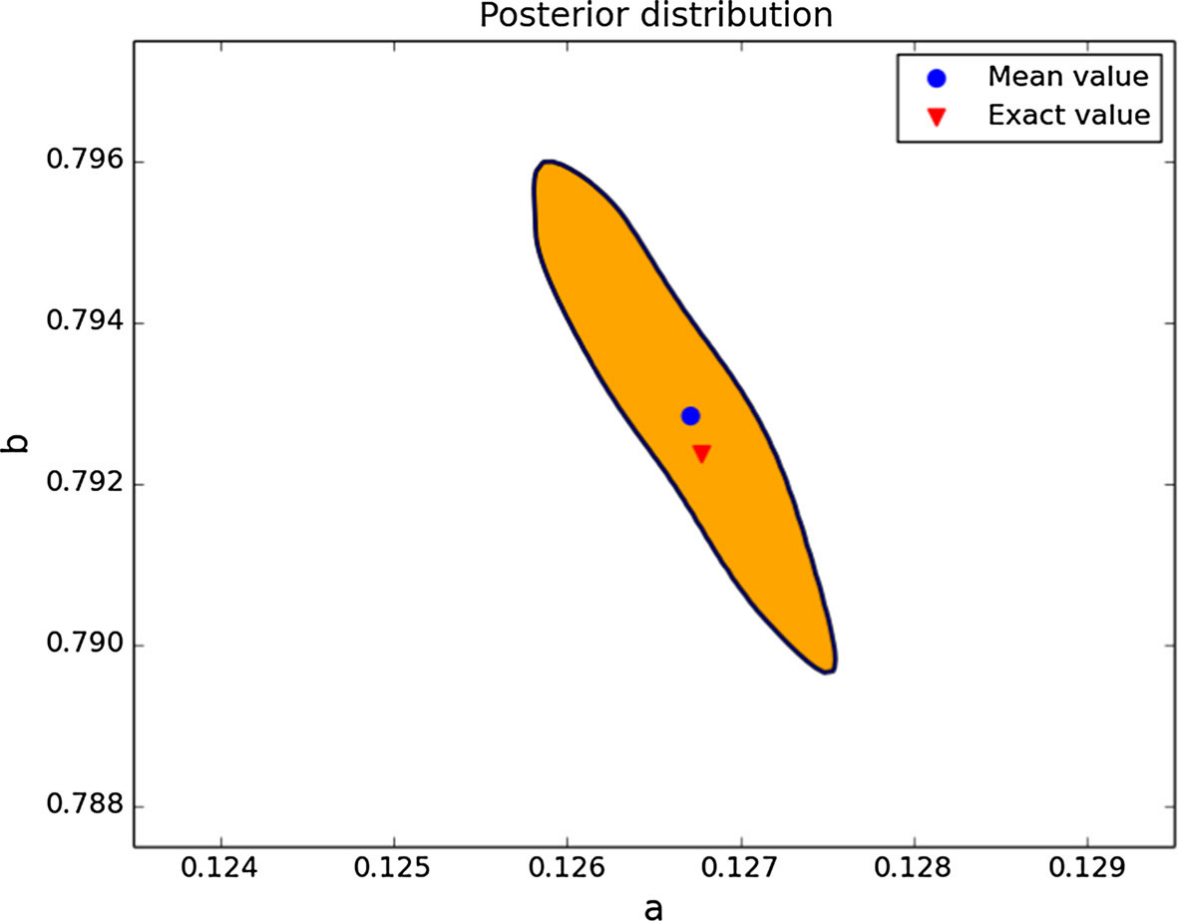
95% credible region for the posterior distribution for the parameters *a* and *b*, using a uniform prior on the region $[0.1,10]^2$. Note that for ease of visualisation, scales for *a* and *b* are different (Color figure online)

For our first example, we assume that the values of the parameters $\gamma$ and *d* are known and that we would like to find the values for the parameters *a* and *b*. In a first approach, we assume very little knowledge about *a* and *b*: only their order of magnitude is assumed to be known. This knowledge is modelled by a uniform prior distribution on the region given by $[0.1,10]^2$. The data for this example have Gaussian noise with standard deviation 5% of the range of the solution. We can see in Fig. 4 a 95% probability region for the posterior distribution. Observe how this region is concentrated around the exact value, in contrast to our original knowledge of a uniform distribution on the region $[0.1,10]^2$. More precisely, the credible region is contained within the range $[0.126,0.128]\times [0.789,0.796]$. The length of the credible region in the *b*-direction is approximately 3.5 times larger than in the *a*-direction, although the size relative to the magnitude of the parameters is smaller in the *b*-direction.

**Fig. 5:**
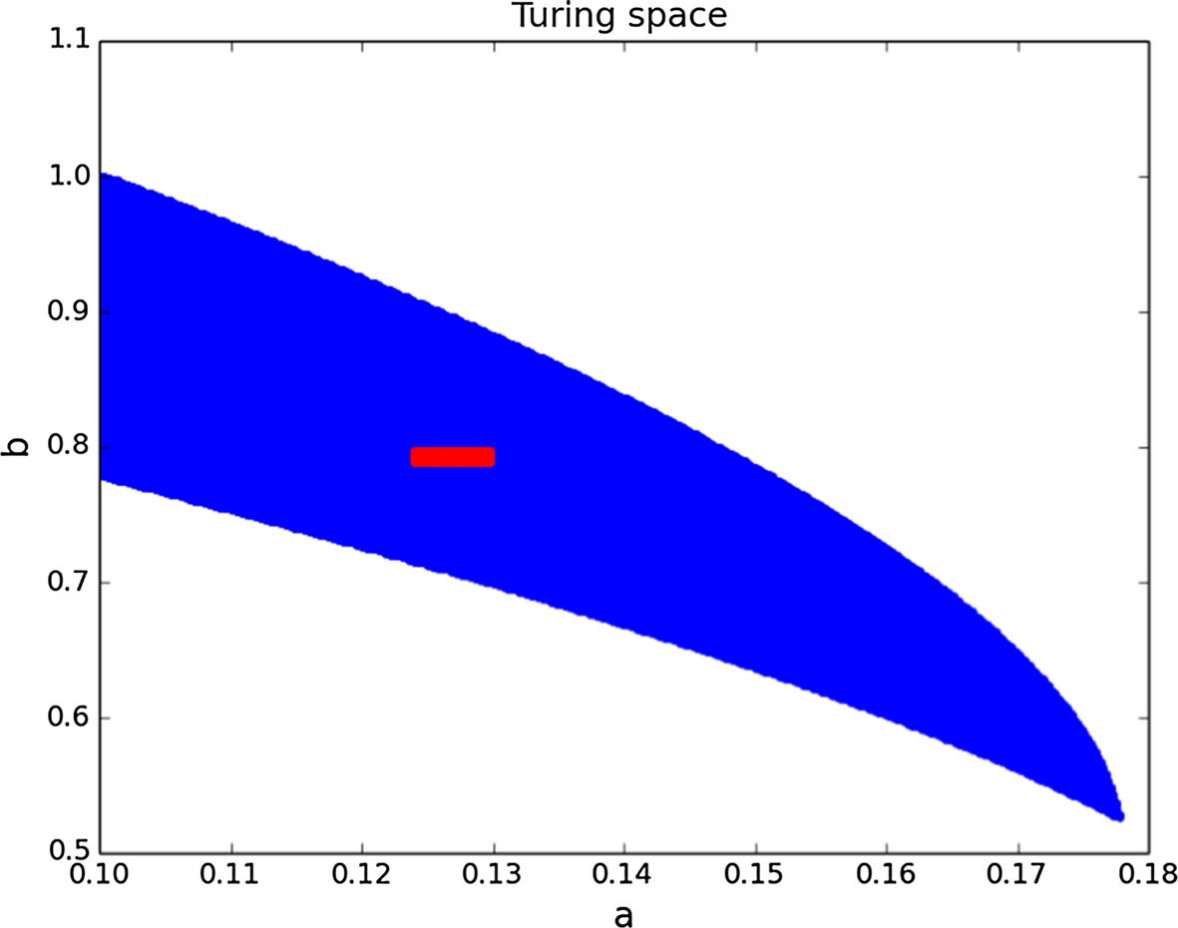
Darker region (blue in the online version) is the Turing space for the parameters *a* and *b* of the Schnakenberg reaction kinetics (Gierer and Meinhardt 1972; Prigogine and Lefever 1968; Schnakenberg 1979). Lighter region (red in the online version) is the region plotted in Fig. 4, depicting where the credible region is contained (Color figure online)

For the Schnakenberg reaction kinetics (Gierer and Meinhardt 1972; Prigogine and Lefever 1968; Schnakenberg 1979), it is possible to compute the region that contains the parameters that can lead to non-homogeneous steady states, the Turing space (see next example for details). We can see that our original prior covered an area much larger than the Turing space (almost 100 times larger), but the posterior is concentrated in a small region completely contained within it (see Fig. 5).

#### Case 2: Credible Regions for *a* and *b* Using the Turing Parameter Space

Unlike the previous example, where we assume little knowledge of the prior, here we exploit the well-known theory for reaction–diffusion systems on stationary domains and use a much more informed prior based on analytical theory of the reaction–diffusion system. On stationary domains, diffusion-driven instability theory requires reaction–diffusion systems to be of the form of *long-range inhibition, short-range activation* for patterning to occur (i.e. $d>1$). More precisely, a necessary condition for Turing pattern formation is that the parameters belong to a well-defined parameter space (Murray 2013) described by the inequalities [for the case of reaction kinetics (4)]

9 $$\begin{aligned} f_u+g_v= & {} \frac{b-a}{b+a} - (a+b)^2 < 0, \nonumber \\ f_u g_v - f_v g_u= & {} (a+b)^2> 0, \nonumber \\ d f_u + g_v= & {} d\left( \frac{b-a}{b+a} \right) - (a+b)^2> 0, \nonumber \\&\qquad (d f_u + g_v)^2 - 4d(f_u g_v - f_v g_u) \nonumber \\= & {} \left( d \left( \frac{b-a}{b+a} \right) - (a+b)^2 \right) ^2 - 4d(a+b)^2 > 0, \end{aligned}$$

where $f_u$, $f_v$, $g_u$ and $g_v$ denote the partial derivatives of *f* and *g* with respect to *u* and *v*, evaluated at the spatially homogeneous steady state $(a+b,\frac{b}{(a+b)^2})$. In Fig. 5 we plot the parameter space obtained with Schnakenberg kinetics.

**Fig. 6:**
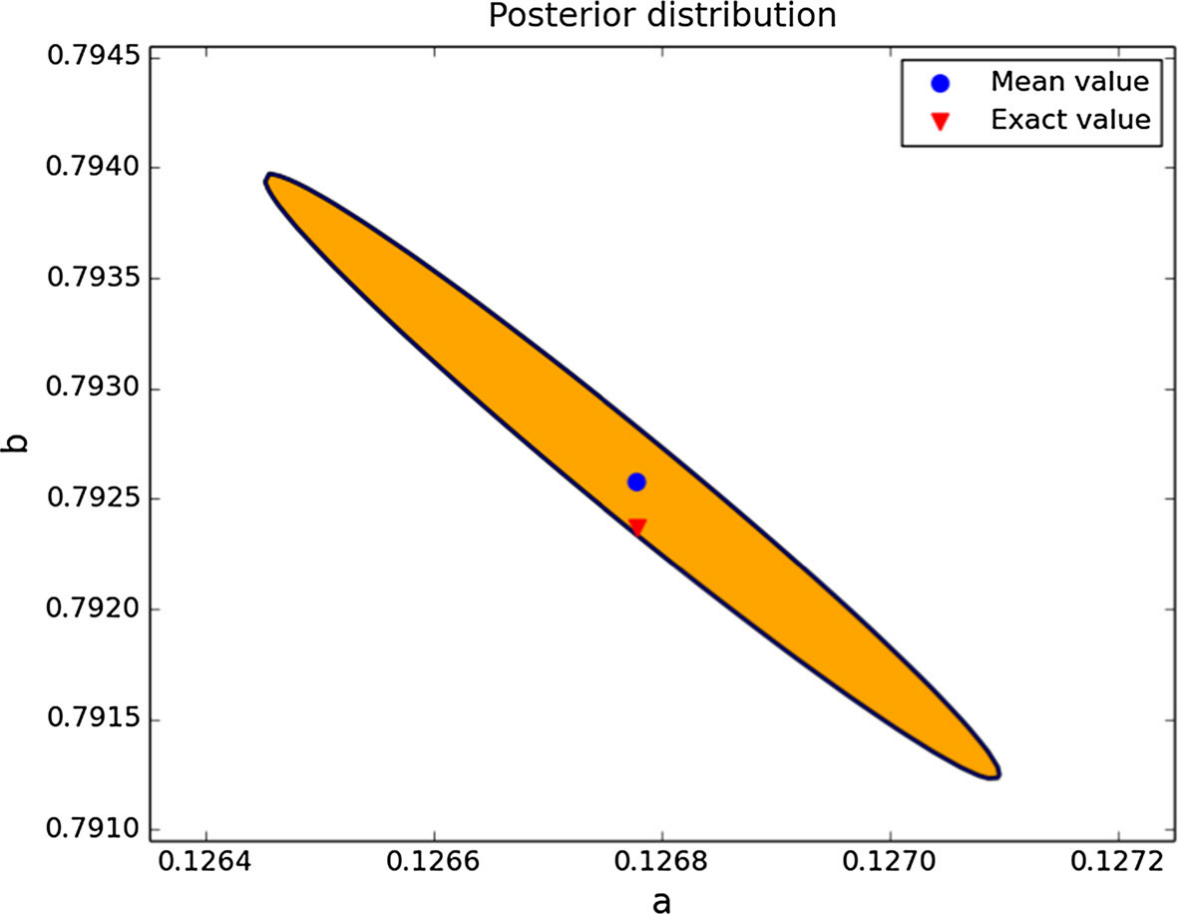
95% credible region for the posterior distribution for the parameters *a* and *b*, using a uniform prior on the Turing space (Color figure online)

In this second example, we use data with added Gaussian noise with standard deviation 10% of the solution range. For the prior, we now use a uniform distribution on the Turing space of the system. In Fig. 6 we can now see that despite the increased noise, the improved prior reduced the size of the 95% probability region dramatically.

#### Case 3: Credible Regions for *d* and $\gamma$

**Fig. 7:**
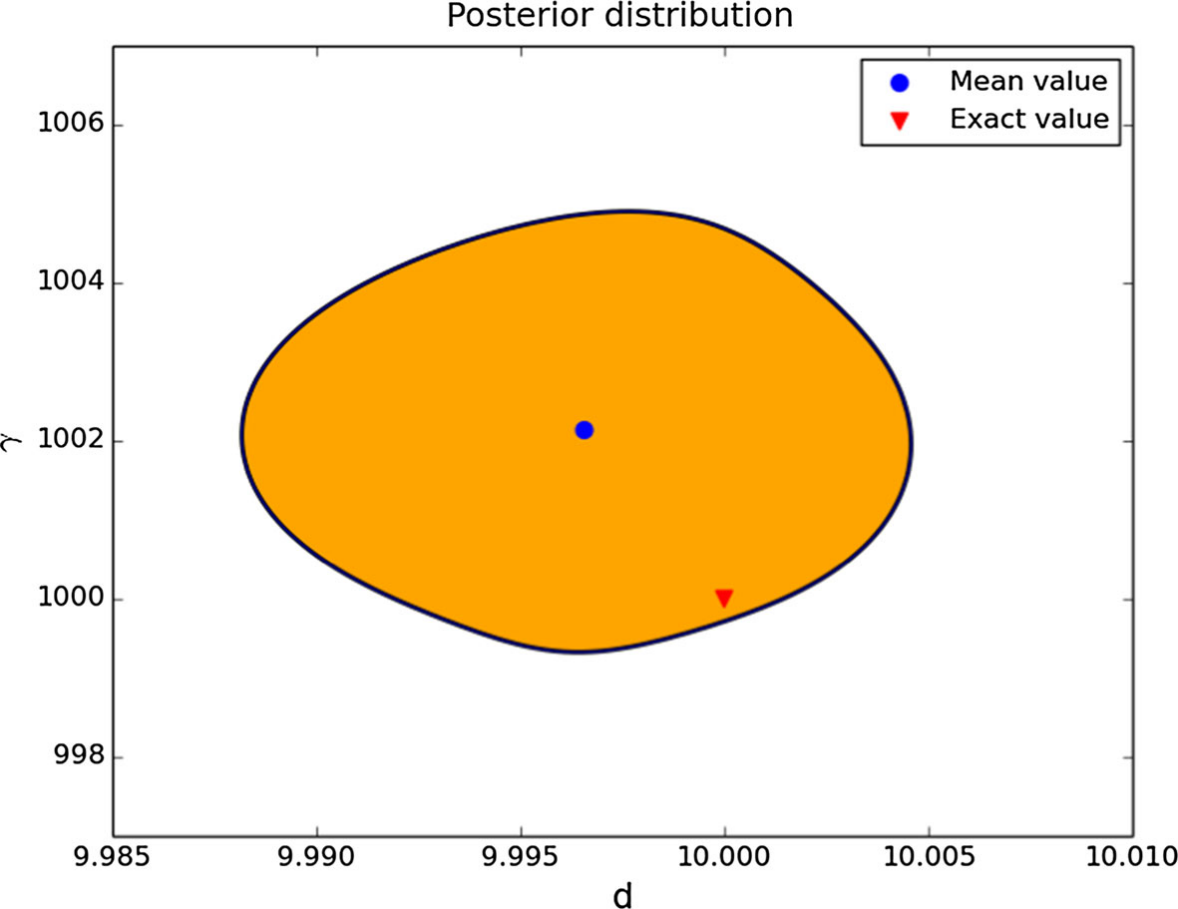
95% credible region for the posterior distribution for the parameters *d* and $\gamma$, a log-normal prior with mean (5, 500) and standard deviation 0.95 (Color figure online)

In a third example, we assume that *a* and *b* are known, and we would like to find $\gamma$ and *d*. To illustrate the use of different types of priors, here we assume a log-normal prior that ensures positivity of $\gamma$ and *d*, which in the case of *d* is necessary to ensure a well-posed problem. We use the data with 5% noise and the prior distribution of a log-normal with mean (5, 500) and standard deviation 0.95. A 95% probability region of the posterior is depicted in Fig. 7. Note that the use of a log-normal or similar prior is essential here to ensure positivity of the diffusion coefficient which is required for the well-posedness of the forward problem. The prior distribution covers a range of one order of magnitude for each parameter, and the posterior distribution suggests relative errors of order $10^{-3}$.

#### Case 4: Credible Regions for *a*, *b*, *d* and $\gamma$

Finally, we identify all four parameters *a*, *b*, *d* and $\gamma$ from the set of data with $10\%$ noise, respectively. We use the priors from *Case 1* for *a* and *b*, and from *Case 3* for *d* and $\gamma$. We do not apply any further restrictions.

**Fig. 8:**
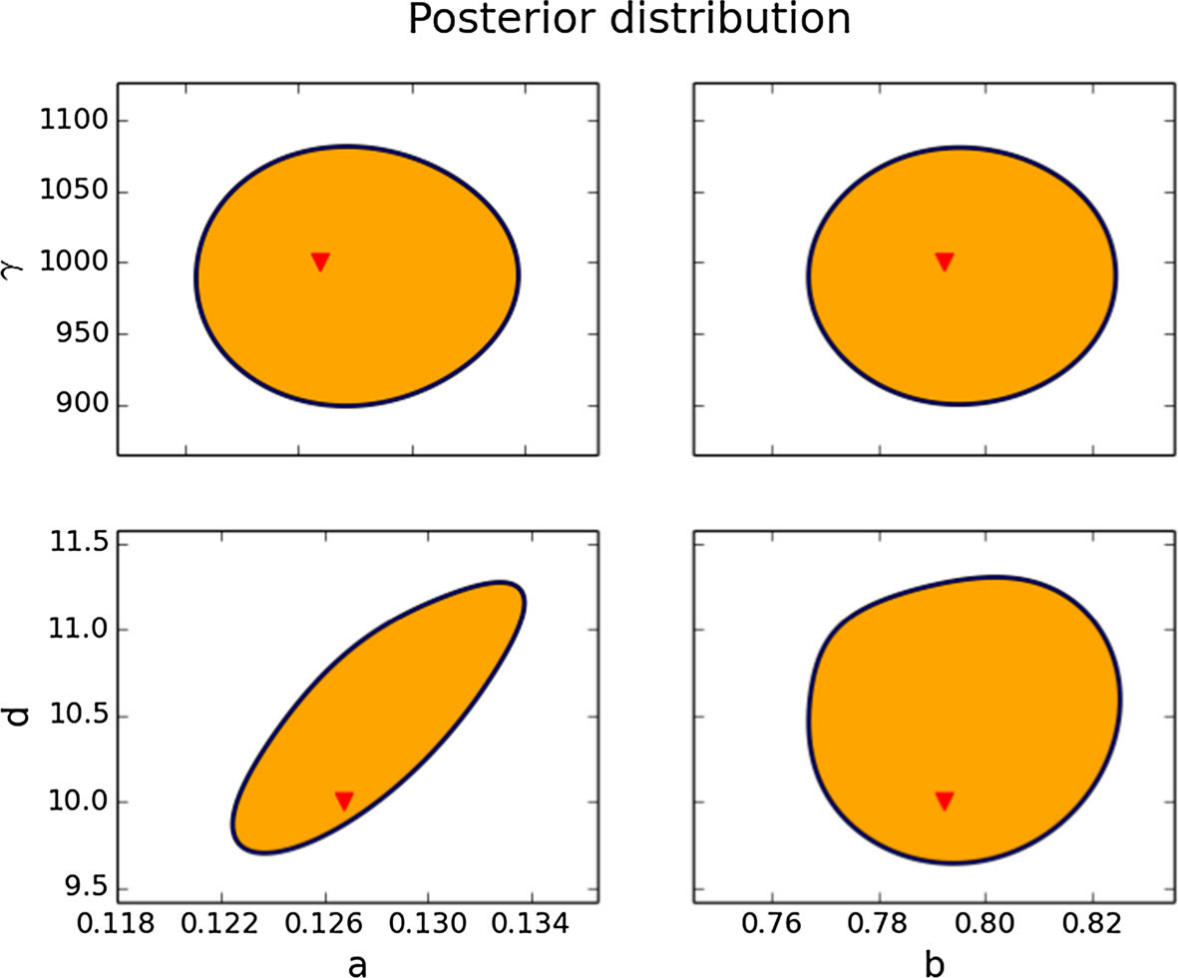
95% credible region for the posterior distribution for the parameters *a*, *b*, *d* and $\gamma$. See *Cases 1* and *3* for a description of the priors. The data have a noise of $10\%$ of the range of the solution. Each subplot corresponds to the projection of the credible region onto a coordinate plane for two of the parameters, given by the row and the column of the subplot. The exact value of the parameters is marked with a triangle (Color figure online)

In Fig. 8 we depict the credible regions for the projection of the parameters to four different coordinate planes. The noise for this experiment is higher than in *Cases 1* and *3*. Also note that compared to the previous experiments we assume here less knowledge *a priori* about the parameters. It must be noted that assuming that a parameter is known is equivalent in the Bayesian formulation to use a Dirac delta for the prior on the parameter; in comparison, the prior distributions for *Case 4* are more spread; therefore, they represent less knowledge. Since the level of noise is higher, and the prior knowledge is lower, the credible regions that we obtain are larger. In this case, the relative errors are of order $10^{-2}$–$10^{-1}$.

## Conclusion

The Bayesian framework offers a mathematically rigorous approach that allows for the inclusion of prior knowledge about the parameters (or more generally functions). Furthermore, the well-posedness results for the identification problem are often possible to obtain under minimal assumptions other than those needed to ensure the well-posedness of the forward problem.

Although exploring a whole probability distribution can be computationally expensive, very useful information about the uncertainty or correlation of the parameters can be inferred from it. The use of a parallel Metropolis–Hastings algorithm makes the computations feasible using HPC facilities.

We studied the parameter identification problem for reaction–diffusion systems using Turing patterns as data for both scenarios: infinite- and finite-dimensional parameter identification cases. We provided a rigorous proof for the well-posedness of the parameter identification problem in the Bayesian sense, and we performed several numerical simulations to find credible regions for the parameters. In particular, we provided numerical evidence that we can distinguish between different growth functions based solely on noisy observations of the data at the end time.

### Further Work

This approach opens many possibilities in the study of inverse problems connected with Turing patterns. As mentioned in Sect. 3.1.2, the initial conditions are not known in a real application with experimental data and can be treated as a parameter as well.

We can also study qualitative properties of the patterns, by means of alternative definitions of the noise and the likelihood. For example, we could take data on the Fourier transform of the pattern in order to identify parameters for different modes—i.e. spots, stripes—in the patterns.

We used synthetic data in this work, but we presented the method keeping in mind the applicability for problems with experimental data. Furthermore, with an adequate PDE solver, we can also apply this approach to problems posed on manifolds. A manuscript presenting the application of these methods with experimental data is in preparation.

A comparison between different methods to estimate posterior distributions in the context of problems involving PDEs and infinite-dimensional parameter would provide more insights into the performance of different algorithms. For example, we could compare the method presented here with approximate Bayesian computation.

The comparison of the approach presented here with the optimal control approach would allow a better understanding of the trade-off between the information about the parameters and the computational cost of the method.

## References

[CR1] Adby P (2013). Introduction to optimization methods.

[CR2] Ashyraliyev M, Jaeger J, Blom JG (2008). Parameter estimation and determinability analysis applied to $$Drosophila$$ gap gene circuits. BMC Syst Biol.

[CR3] Ashyraliyev M, Fomekong-Nanfack Y, Kaandorp JA, Blom JG (2009). Systems biology: parameter estimation for biochemical models. FEBS J.

[CR4] Aster RC, Borchers B, Thurber CH (2013). Parameter estimation and inverse problems.

[CR5] Battogtokh D, Asch DK, Case ME, Arnold J, Schüttler HB (2002). An ensemble method for identifying regulatory circuits with special reference to the qa gene cluster of $$Neurospora crassa$$. Proc Natl Acad Sci.

[CR6] Beck JV, Blackwell B, St. Clair CR Jr (1985) Inverse heat conduction: Ill-posed problems. Wiley, New York

[CR7] Blazakis KN, Madzvamuse A, Reyes-Aldasoro CC, Styles V, Venkataraman C (2015). Whole cell tracking through the optimal control of geometric evolution laws. J Comput Phys.

[CR8] Brown KS, Sethna JP (2003). Statistical mechanical approaches to models with many poorly known parameters. Phys Rev E.

[CR9] Calderhead B (2014). A general construction for parallelizing Metropolis–Hastings algorithms. Proc Natl Acad Sci.

[CR10] Cotter SL, Roberts GO, Stuart AM, White D (2013). MCMC methods for functions: modifying old algorithms to make them faster. Stat Sci.

[CR11] Crampin E, Hackborn W, Maini P (2002). Pattern formation in reaction–diffusion models with nonuniform domain growth. Bull Math Biol.

[CR12] Crampin EJ, Gaffney EA, Maini PK (1999). Reaction and diffusion on growing domains: scenarios for robust pattern formation. Bull Math Biol.

[CR13] Croft W, Elliott C, Ladds G, Stinner B, Venkataraman C, Weston C (2015). Parameter identification problems in the modelling of cell motility. J Math Biol.

[CR14] Das D (2017). Turing pattern formation in anisotropic medium. J Math Chem.

[CR15] Dashti M, Stuart AM (2013) The Bayesian approach to inverse problems. arXiv:1302.6989

[CR16] Fan W, Bouguila N, Ziou D (2012). Variational learning for finite Dirichlet mixture models and applications. IEEE Trans Neural Netw Learn Syst.

[CR17] Friedman A, Reitich F (1992). Parameter identification in reaction–diffusion models. Inverse Probl.

[CR18] Garvie MR, Trenchea C (2014). Identification of space–time distributed parameters in the Gierer–Meinhardt reaction–diffusion system. SIAM J Appl Math.

[CR19] Garvie MR, Maini PK, Trenchea C (2010). An efficient and robust numerical algorithm for estimating parameters in turing systems. J Comput Phys.

[CR20] Gierer A, Meinhardt H (1972). A theory of biological pattern formation. Kybernetik.

[CR21] Guiu-Souto J, Muñuzuri AP (2015). Influence of oscillatory centrifugal forces on the mechanism of turing pattern formation. Phys Rev E.

[CR22] Gutenkunst RN, Waterfall JJ, Casey FP, Brown KS, Myers CR, Sethna JP (2007). Universally sloppy parameter sensitivities in systems biology models. PLoS Comput Biol.

[CR23] Hu X, Liu Y, Xu X, Feng Y, Zhang W, Wang W, Song J, Wang Y, Zhao W (2015). Spatiotemporal evolution of a cosine-modulated stationary field and Kerr frequency comb generation in a microresonator. Appl Opt.

[CR24] Huttunen JM, Kaipio JP (2007). Approximation errors in nonstationary inverse problems. Inverse Probl Imaging.

[CR25] Iglesias MA, Lin K, Stuart AM (2014). Well-posed Bayesian geometric inverse problems arising in subsurface flow. Inverse Probl.

[CR26] Jones E, Oliphant T, Peterson P et al (2001) SciPy: open source scientific tools for python

[CR27] Kaipio J, Somersalo E (2006). Statistical and computational inverse problems.

[CR28] Kaipio J, Somersalo E (2007). Statistical inverse problems: discretization, model reduction and inverse crimes. J Comput Appl Math.

[CR29] Lacitignola D, Bozzini B, Frittelli M, Sgura I (2017). Turing pattern formation on the sphere for a morphochemical reaction–diffusion model for electrodeposition. Commun Nonlinear Sci Numer Simul.

[CR30] Lakkis O, Madzvamuse A, Venkataraman C (2013). Implicit-explicit timestepping with finite element approximation of reaction–diffusion systems on evolving domains. SIAM J Numer Anal.

[CR31] Ma Z, Leijon A (2011). Bayesian estimation of beta mixture models with variational inference. IEEE Trans Pattern Anal Mach Intell.

[CR32] Ma Z, Teschendorff AE, Leijon A, Qiao Y, Zhang H, Guo J (2015). Variational bayesian matrix factorization for bounded support data. IEEE Trans Pattern Anal Mach Intell.

[CR33] Mackenzie J, Madzvamuse A (2011). Analysis of stability and convergence of finite-difference methods for a reaction–diffusion problem on a one-dimensional growing domain. IMA J Numer Anal.

[CR34] Madzvamuse A (2006). Time-stepping schemes for moving grid finite elements applied to reaction–diffusion systems on fixed and growing domains. J Comput Phys.

[CR35] Madzvamuse A, Maini PK (2007). Velocity-induced numerical solutions of reaction–diffusion systems on continuously growing domains. J Comput Phys.

[CR36] Madzvamuse A, Maini P, Wathen A (2005). A moving grid finite element method for the simulation of pattern generation by turing models on growing domains. J Sci Comput.

[CR37] Madzvamuse A, Gaffney EA, Maini PK (2010). Stability analysis of non-autonomous reaction–diffusion systems: the effects of growing domains. J Math Biol.

[CR38] Madzvamuse A, Ndakwo HS, Barreira R (2016). Stability analysis of reaction–diffusion models on evolving domains: the effects of cross-diffusion. Dyn Syst.

[CR39] Murray JD (2011). Mathematical biology: I. An introduction.

[CR40] Murray JD (2013). Mathematical biology II: spatial models and biomedical applications.

[CR41] Norris JR (1998). Markov chains.

[CR42] Perdikaris P, Karniadakis GE (2016). Model inversion via multi-fidelity Bayesian optimization: a new paradigm for parameter estimation in haemodynamics, and beyond. J R Soc Interface.

[CR43] Portet S, Madzvamuse A, Chung A, Leube RE, Windoffer R (2015) Keratin dynamics: modeling the interplay between turnover and transport. PLoS ONE. 10.1371/journal.pone.0121090 10.1371/journal.pone.0121090 PMC4379186 25822661

[CR44] Prigogine I, Lefever R (1968). Symmetry breaking instabilities in dissipative systems. II. J Chem Phys.

[CR45] Ross RJ, Baker RE, Parker A, Ford M, Mort R, Yates C (2017). Using approximate bayesian computation to quantify cell–cell adhesion parameters in a cell migratory process. NPJ Syst Biol Appl.

[CR46] Ruuth S (1995). Implicit–explicit methods for reaction–diffusion problems in pattern formation. J Math Biol.

[CR47] Schnakenberg J (1979). Simple chemical reaction systems with limit cycle behaviour. J Theor Biol.

[CR48] SIAM Working Group on CSE SWGoC (2001). Graduate education in computational science and engineering. SIAM Rev.

[CR49] Stoll M, Pearson JW, Maini PK (2016). Fast solvers for optimal control problems from pattern formation. J Comput Phys.

[CR50] Stuart AM (2010). Inverse problems: a Bayesian perspective. Acta Numer.

[CR51] Sutton JE, Guo W, Katsoulakis MA, Vlachos DG (2016). Effects of correlated parameters and uncertainty in electronic-structure-based chemical kinetic modelling. Nat Chem Adv.

[CR52] Tarantola A (2005) Inverse problem theory and methods for model parameter estimation. Society for Industrial and Applied Mathematics, Other Titles in Applied Mathematics

[CR53] Tierney L (1998). A note on Metropolis–Hastings kernels for general state spaces. Ann Appl Probab.

[CR54] Tjelmeland H (2004) Using all Metropolis–Hastings proposals to estimate mean values. Norwegian University of Science and Technology, Trondheim, Norway. Tech Rep 4

[CR55] Toni T, Welch D, Strelkowa N, Ipsen A, Stumpf MP (2009). Approximate Bayesian computation scheme for parameter inference and model selection in dynamical systems. J R Soc Interface.

[CR56] Turing AM (1952). The chemical basis of morphogenesis. Philos Trans R Soc Lond B Biol Sci.

[CR57] Uzunca M, Küçükseyhan T, Yücel H, Karasözen B (2017). Optimal control of convective Fitzhugh–Nagumo equation. Comput Math Appl.

[CR58] Venkataraman C, Lakkis O, Madzvamuse A (2012). Global existence for semilinear reaction–diffusion systems on evolving domains. J Math Biol.

[CR59] Venkataraman C, Lakkis O, Madzvamuse A, Cangiani A, Davidchack R, Georgoulis E, Gorban A, Levesley J, Tretyakov M (2013). Adaptive finite elements for semilinear reaction-diffusion systems on growing domains. Numerical mathematics and advanced applications 2011.

[CR60] Vigil RD, Ouyang Q, Swinney HL (1992). Turing patterns in a simple gel reactor. Physica A Stat Mech Appl.

[CR61] Vyshemirsky V, Girolami MA (2008). Bayesian ranking of biochemical system models. Bioinformatics.

[CR62] Wang J, Zhang M, Li M, Wang Y, Liu D (2016) On the control of the microresonator optical frequency comb in turing pattern regime via parametric seeding. In: OptoElectronics and communications conference (OECC) held jointly with 2016 international conference on photonics in switching (PS), 2016 21st, IEEE, pp 1–3

[CR63] Xun X, Cao J, Mallick B, Maity A, Carroll RJ (2013). Parameter estimation of partial differential equation models. J Am Stat Assoc.

[CR64] Yang F, Venkataraman C, Styles V, Madzvamuse A (2015) A parallel and adaptive multigrid solver for the solutions of the optimal control of geometric evolution laws in two and three dimensions. In: 4th international conference on computational and mathematical biomedical engineering—CMBE2015, pp 1–4

